# Fly Reservoir Associated with *Wohlfahrtiimonas* Bacteremia in a Human

**DOI:** 10.3201/eid2402.170913

**Published:** 2018-02

**Authors:** Jesse H. Bonwitt, Michael Tran, Elizabeth A. Dykstra, Kaye Eckmann, Melissa E. Bell, Michael Leadon, Melissa Sixberry, William A. Glover

**Affiliations:** Washington State Department of Health, Shoreline, Washington, USA (J.H. Bonwitt);; University of Durham, Durham, United Kingdom (J.H. Bonwitt);; Centers for Disease Control and Prevention, Atlanta, Georgia, USA (J.H. Bonwitt, M.E. Bell);; Washington State Public Health Laboratories, Shoreline, Washington, USA (M. Tran, K. Eckmann, W.A. Glover);; Washington State Department of Health, Olympia, Washington, USA (E.A. Dykstra);; Yakima Health District, Yakima, Washington, USA (M. Leadon, M. Sixberry)

**Keywords:** Calliphoridae, green bottle fly, Lucilia sericata, Diptera, pulsed-field gel electrophoresis, larvae, MALDI-TOF, mass spectrometry, myiasis, 16S ribosomal RNA, Wohlfahrtiimonas, bacteria, bacteremia, Washington, United States

## Abstract

*Wohlfahrtiimonas* species bacteria were isolated from the bloodstream of a patient with septicemia and wound myiasis. Environmental investigations identified a *Wohlfahrtiimonas* sp. among insects in the Americas and in a previously undescribed vector, the green bottle fly (*Lucilia sericata*). The isolates possibly represent a new species within the genus *Wohlfahrtiimonas*.

*Wohlfahrtiimonas chitiniclastica* is a rarely reported cause of bacterial infection that has been isolated in humans and other mammals from a variety of organs (Technical Appendix Table). In addition, *Wohlfahrtiimonas* spp. have been isolated from 4 species of nonbiting flies in Asia and Europe (*1**–**4*) that can cause myiasis, fly larvae infestation of a host’s tissue. Wound myiasis has been reported in patients infected with *W. chitiniclastica* and with *Ignatzschineria* spp., an organism closely related to *W. chitiniclastica* (Technical Appendix Table). These findings provide evidence that *W. chitiniclastica* is transmitted by flies or fly larvae during myiasis. However, no reported attempt has been made to isolate *Wohlfahrtiimonas* spp. or *Ignatzschineria* spp. from larvae associated with a patient. We report a case of *Wohlfahrtiimonas* infection in a man in Washington, USA, and results of environmental investigations.

## The Study

The case-patient was a 57-year-old man who developed wet gangrene of the right ankle and myiasis below the waist. Hematology at hospital admission was notable for leukocytosis and a predominance of neutrophils with a high ratio of band neutrophils (Technical Appendix). Chronic cirrhosis, localized lung atelectasis, and multiorgan failure secondary to septic shock were diagnosed. The patient underwent amputation below the right knee but died 3 days after admission. Blood, urine, and tracheal aspirates collected <8 hours after admission revealed a mixed bacterial infection, including gram-positive cocci and gram-negative rods (Technical Appendix). *Propionibacterium acnes* and *Staphylococcus hominis* ssp. *hominis* were isolated from blood cultures, in addition to an unidentifiable gram-negative rod. No medical history was available; proxy interviews excluded recent travel outside Washington.

We performed presumptive identification of the gram-negative rod with phenotypic studies and matrix-assisted laser desorption/ionization time-of-flight mass spectrometry (Technical Appendix). Amplification and sequencing of the near full-length 16S ribosomal RNA (rRNA) gene was performed, a phylogenetic tree was inferred by using the neighbor-joining method, and the topology was assessed by a bootstrap analysis of 1,000 replicates (Technical Appendix). We used pulsed-field gel electrophoresis (PFGE) to assess isolate relatedness (Technical Appendix).

Because larvae found on the patient had been discarded, we collected live and dead insects from the patient’s home and identified them to genus or species level (Technical Appendix). To remove surface contamination, all live fly larvae and adult specimens were rinsed 5 times with sterile phosphate-buffered saline (PBS), homogenized, and sequentially diluted. We cultured the first rinse, fifth rinse, and diluted homogenates to isolate *Wohlfahrtiimonas* spp. (Technical Appendix).

**Figure 1:**
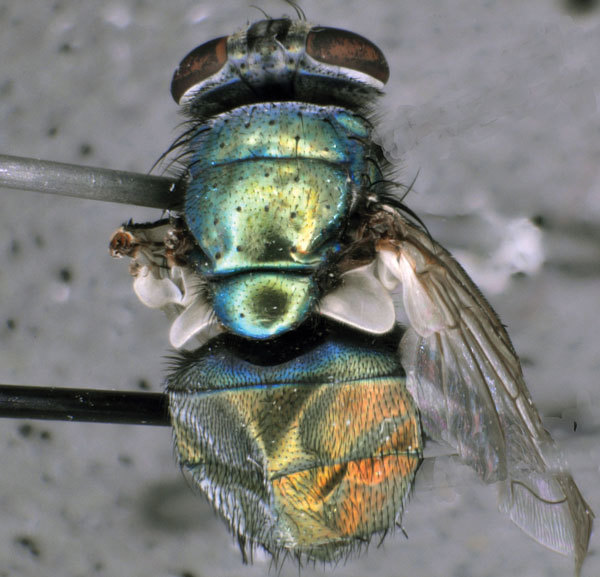
Green bottle fly (*Lucilla sericata*), caught inside home of patient with septicemia and wound myiasis in Washington, USA. The fly laid eggs inside a sterile container, and a *Wohlfahrtiimonas* spp. were isolated from a larva hatched from these eggs. Photo courtesy of T. Whitworth.

We identified 6 species of flies (Technical Appendix) and collected live larvae (≈20) from the patient’s house (Table, batch 1). We performed bacterial culture on a pooled sample of half of these larvae (Table, sample 2) and then individually on adult flies that emerged from the other half (Table, samples 3–5). One green bottle fly (*Lucilia sericata*) (Figure 1) was caught alive in the house in a sterile container and laid eggs inside the container before dying (Table, batch 2). We isolated a *Wohlfahrtiimonas* sp. from 2 of 6 insect samples on blood agar plates (Table, samples 2–7) but not from any other samples, including adult flies that emerged from the positive batch of larvae.

**Table:** Culture results for *Wohlfahrtiimonas* spp. from samples collected from patient with septicemia and wound myiasis and the patient’s home, Washington, USA*

Collection batch no.	Sample no.	Specimen	Specimen description	Culture *Wohlfahrtiimonas* spp.
NA	1	Blood	Isolate sent to public health laboratories from admitting hospital	Aerobic growth on blood agar (isolate 22912)
1	2	Fly larvae (unidentified species, n ≈ 20)	Larvae collected from underneath carpet where patient was found	Growth on diluted homogenate on blood agar at 25°C (isolate 22913)
1	3	House fly (*Musca domestica*)	Emerged from larvae of sample no. 2	No growth
1	4	Unidentified species in the family Calliphoridae	Emerged from larvae of sample no. 2	No growth
1	5	*Calliphora vicina*	Emerged from larvae of sample no. 2	No growth
2	6	Green bottle fly and eggs (*Lucilla sericata*)	Green bottle fly caught inside the patient’s home and laid eggs inside a sterile container	Not cultured
2	7	*Lucilla sericata* larva	Larva obtained from the egg in batch no. 2	Growth on fifth wash (isolate 22914) and diluted homogenate (isolate 22915) on blood agar at 25°C
NA	8	Meat and fruit	Fed to flies from samples 3–7 were extracted	No growth

*NA, not applicable

**Figure 2:**
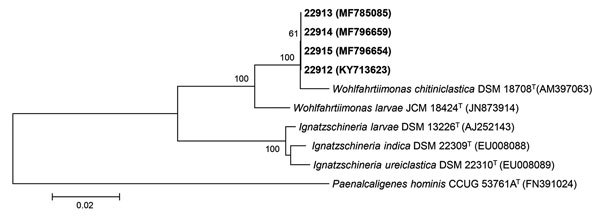
Neighbor-joining phylogenetic tree of 16S rRNA gene sequences of *Wohlfahrtiimonas* spp. isolate from a patient with septicemia and wound myiasis in Washington, USA (laboratory identification no. 22912), isolates from flies and fly larvae (laboratory identification nos. 22913, 22914, 22915), and the most closely related type strains. Numbers at nodes denote bootstrap percentages based on 1,000 replicates; only values >50% are shown. GenBank accession numbers are given in parentheses. Bold indicates strains isolated in this study. The tree was rooted with *Paenalcaligenes hominis* CCUG 53761A^T^ as the outgroup. Scale bar indicates substitutions per nucleotide position.

The isolates grew on blood agar, yielding colonies with a smooth center and rough edges, and displayed α hemolysis. Matrix-assisted laser desorption/ionization time-of-flight mass spectrometry yielded a presumptive result of *W. chitiniclastica* (Technical Appendix). A phylogenetic analysis of the 16S rRNA gene sequence of all isolates (1,462 bp; see Figure 2 for GenBank accession numbers) showed that the most closely related type strains were *W. chitiniclastica* DSM 18708^T^ (98.3% sequence similarity) and *W. larvae* JCM 18424^T^ (97.3% sequence similarity) (Figure 2). The PFGE pattern indicated that all isolates from flies and fly larvae were indistinguishable and 74% similar to that of the patient isolate (Technical Appendix).

## Conclusions

Our isolates possibly represent a new species in the genus *Wohlfahrtiimonas* based on the percentage sequence similarity with *W. chitiniclastica* and *W. larvae* type strains (*5*). We isolated *Wohlfahrtiimonas* sp. from insects in the Americas and in a previously undescribed host, the green bottle fly (*L. sericata*, Diptera: Calliphoridae). Previously, *Wohlfahrtiimonas* spp. have been identified in only 4 species of flies in Asia and Europe (*Wohlfahrtia magnifica, Chrysomya megacephala*, *Hemetia illucens*, *Musca domestica*) (*1**–**4*), each representing a different fly family (Diptera: Sarcophagidae, Calliphoridae, Stratiomyidae, and Muscidae, respectively). We isolated a *Wohlfahrtiimonas* from a larva hatched from eggs laid by a fly in a sterile container, providing evidence that *Wohlfahrtiimonas* sp. can be transmitted vertically.

*L. sericata* has been associated with *W. chitiniclastica* infection in a patient with myiasis and bacteremia only once, in the United Kingdom (*6*), but a definitive link could not be established in that case because larvae from the patient had been discarded. The scarcity of reports of *Wohlfahrtiimonas* spp. infections might be attributable to the difficulty in laboratory identification (*7**,**8*) or because wound myiasis is routinely addressed with broad-spectrum antimicrobial drugs.

Because the pooled larvae (Table, sample 2) emerged as multiple fly species, we are unable to ascertain in which other species *Wohlfahrtiimonas* sp. growth occurred. *Wohlfahrtiimonas* was not isolated from these adult flies, which might be because the competent host was not present in the batch of larvae left to emerge or because of the association between *Wohlfahrtiimonas* spp. and flies during successive developmental stages. Indeed, previous studies isolated *W. chitiniclastica* from the gut of larvae and adult flies (*1**,**3**,**4*), and *Ignatzschineria* spp. are hypothesized to play a role in larval development (*9*), indicating that these bacteria might belong to fly microbiota. In one study, the relative abundance of *Ignatzschineria* spp. fluctuated during life stages of *L. sericata* and was among the dominant bacterial genera during the larval and pupal life stages (*10*). Bacterial flora further decline during pupation, when reorganization of the intestinal tract leads to extrusion of the gut lining (*11*). These factors might explain why we did not isolate *Wohlfahrtiimonas* spp. from the adult flies that emerged, or our protocol might have been of insufficient diagnostic sensitivity to detect *Wohlfahrtiimonas* spp. among adult flies.

The concurrent isolation of *Wohlfahrtiimonas* sp. in the blood from a patient with myiasis and in fly larvae found at the patient’s home provides further evidence that fly larvae can act as vectors of *Wohlfahrtiimonas* spp. Because PFGE patterns of the isolates obtained from the fly larvae and from the patient’s blood did not match, we cannot definitely identify the fly species that led to his infection.

We isolated *Wohlfahrtiimonas* sp. from a patient’s blood along with other bacteria, precluding us from assessing the pathogenicity of our isolate. However, in 2 previous reports (*12**,**13*), *W. chitiniclastica* was the only bacterium isolated from the blood, indicating its pathogenic potential (Technical Appendix Table 1).

Most cases of *W. chitiniclastica* infection have occurred among persons with a history of poor hygiene and exposed wounds (Technical Appendix Table 1). Green bottle flies are among the most common species associated with myiasis in the United States (*14*), and risk for infection is expected during warm environmental conditions favorable to their development. In addition, green bottle fly larvae are the most commonly used larvae for maggot debridement therapy (*15*) Infection with *Wohlfahrtiimonas* spp. should be considered as a potential risk for patients undergoing this therapy.

Technical Appendix Additional details on case of isolation of *Wohlfahrtiimonas* spp. from a patient with septicemia and wound myiasis in Washington, USA.
