# Supplementary material for: Fly Reservoir Associated with Wohlfahrtiimonas Bacteremia in a Human
Source: Emerg Infect Dis. 2018 Feb;24(2):370–3. doi: 10.3201/eid2402.170913 (PMC5782874; doi:10.3201/eid2402.170913)
Supplement: Technical Appendix — Additional details on case of isolation of Wohlfahrtiimonas spp. from a patient with septicemia and wound myiasis in Washington, USA. [file 17-0913-Techapp-s1.pdf]

Article DOI: https://doi.org/10.3201/eid2402.170913

Fly Reservoir Associated with *Wohlfahrtiimonas* Bacteremia in a Human

Technical Appendix

One Case Presentation

The case-patient's hematology upon admission was notable for leukocytosis 21.8 K/µL (reference 3.8–11.0 K/uL) and a predominant neutrophil/polymorphonuclear neutrophil ratio of 44.0% (reference 38.0%–70.0%) with a high ratio of band neutrophils, 33.0% (reference 0%–9.0%).

Blood, urine, and tracheal aspirates collected <8 hours after admission revealed a mixed bacterial infection, including gram-positive cocci and gram-negative rods. Blood culture yielded *Propionibacterium acnes* (field draw, anaerobic bottle), *Staphylococcus hominis* ssp. *hominis* and *Wohlfahrtiimonas* sp. (short draw, aerobic bottle only). Tracheal aspirates yielded heavy-growth *Haemophilus influenzae*. The urine culture was negative. *Wohlfahrtiimonas* sp. could not be identified by the referring hospital and the samples were sent for identification to the Reference Bacteriology Laboratory at the Washington State Public Health Laboratories (PHL).

Two Methods

Phenotypic Studies

The isolate obtained from the patient was inoculated on blood agar, MacConkey agar, and heart infusion tyrosine agar at 35°C in aerobic conditions. Phenotypic studies for presumptive identification were performed as previously described (*1*). The isolate was tested for anaerobic growth at 35°C by using an anaerobic gas pack (BD GasPak, Becton Dickinson, Franklin Lakes, NJ, USA) in an anaerobic jar.

MALDI-TOF MS

Isolates were initially analyzed by matrix-assisted laser desorption/ionization time-of-flight mass-spectrometry (MALDI-TOF MS, MALDI Biotyper CA System, Bruker Daltonics,

Billerica, MA, USA) in accordance with the manufacturer's recommendations (*2*). The generated spectra were compared with the spectra referenced in the MALDI-TOF MS database (PHL: RUO library MBT BD-5627 MSP List; CDC: Bruker 6903 database). For analysis, an isolate was considered as identified for log scores >2.0.

16S Ribosomal RNA Sequencing

Purification of whole-cell DNA and amplification and sequencing of the near full-length 16S ribosomal RNA (rRNA) gene were performed at CDC as previously described (*3*). The 16S rRNA gene sequence was assembled with Geneious 8.1.8 software (Biomatters, Auckland, New Zealand) by using de novo assembly. To identify related gene sequences in the GenBank database, consensus sequence was submitted to GenBank by using BLASTN software (https://www.ncbi.nlm.nih.gov/blast/). A multiple sequence alignment was created by using Clustal W (within Geneious 8.1.8), from which gaps and 5′ and 3′ ends were trimmed.

We inferred a phylogenetic tree by using the neighbor-joining method (*4*), and the topology was assessed by a bootstrap analysis of 1000 replicates (*5*). We computed the evolutionary distances by using the Tamura-Nei method (*6*); they are in the units of the number of base substitutions per site. Evolutionary analyses were conducted in MEGA6 (*7*).

Entomology

All larvae found on the patient were discarded before the investigation and could not be sampled, but live and dead insects were collected from the patient's home. Insects were identified to genus or species level by using standard entomological identification keys (*8–10*). Live fly larvae were left to hatch at room temperature inside a mosquito-breeding container (BioQuip Products, Rancho Dominguez, CA, USA). Fly larvae were provided raw meat and freshly boiled chicken; adult flies were provided slices of fresh fruit.

Bacterial Isolation from Insects

Live fly larvae and adults were cultured for *Wohlfahrtiimonas* spp. Because the preferred growth medium of *Wohlfahrtiimonas* spp. is poorly described, all samples were inoculated on 3 replicates of blood agar, MacConkey agar, and nutrient agar. Phenylethyl alcohol agar was used for initial specimens (larvae) obtained from the patient's home. Plates were incubated at 25°C, 30°C, and 35°C in aerobic conditions over 2 days.

To remove surface contamination, we rinsed all insect specimens 5 times with sterile PBS in sterile tubes, as described previously (*11*). We homogenized the washed specimens with a sterile mortar (Fisher Scientific, Richardson, TX, USA) in 0.5 mL PBS and sequentially diluted to 1/1000. The first wash, fifth wash, undiluted homogenate, and diluted homogenate were inoculated in the same manner as fly larvae and adults. The objective was to verify whether surface contamination was present (first wash) and, if so, whether it was removed through rinsing (fifth wash). To verify that live insects were not inoculated with *Wohlfahrtiimonas* spp. through contaminated food, the meat and fruit (with the exception of boiled chicken) was homogenized and cultured in the same manner as fly larvae and adults.

To reduce the risk for agar plates being overgrown by other bacterial species and increase the sensitivity of isolating *Wohlfahrtiimonas* spp., we placed a sample of the diluted homogenate in nutrient broth made with 11.5 g of Difco nutrient agar (Becton Dickinson) and 500 mL distilled water and incubated at 25°C, 30°C, and 35°C over 2 days. We then inoculated this material on agar for all samples that did not initially grow *Wohlfahrtiimonas* spp.

Pulsed-Field Gel Electrophoresis

To assess relatedness, we performed pulsed-field gel electrophoresis (PFGE) on the isolates using CDC's standardized PFGE PulseNet protocol for *Escherichia coli* O157:H7 (*12*). Because PFGE has not been previously described for *Wohlfahrtiimonas* spp., we experimented with restriction enzymes commonly used on gram-negative organisms related to *Wohlfahrtiimonas* spp. (*13,14*), including *Not*I (Roche), *XBa*I (Roche) and *Spe*I (Roche) restriction enzymes. The standard lanes consisted of *Salmonella* ser. Braenderup H9812 cut with restriction enzyme *Xba*I. Gel electrophoresis was run by using CHEF Mapper XA system (Bio-Rad Laboratories, Hercules, CA, USA) for 18.5 hours with initial and final switch time at 2.2 and 54.2 seconds, respectively. Analysis of PFGE patterns was conducted with BioNumerics (version 6.6). *Salmonella*. ser. Braenderup H9812 was used as the reference standard.

Three Results

Case Investigation and Entomological Identification

On September 15, 2016, we inspected the patient's home, which consisted of an urban, single-story detached building in insalubrious condition. The outdoor temperature range was 21°C–27°C. One window was ajar by ≈5cm, allowing insects to enter the home.

We found a substantial number of dead insects in a desiccated state inside the home. We identified 6 species of flies, including *Calliphora vicina* (blue bottle fly), *Lucilia sericata* (green bottle fly), *Musca domestica* (house fly), *Hydrotaea leucostoma* (garbage fly), *Protophormia terranovae* (northern blow fly) and *Fannia* species (lesser house fly). Live larvae (n ≈ 20) were collected from underneath a carpet soiled with organic matter at the exact location where the patient was found. Half of these larvae were kept to perform entomological bacteriology; the other half were left to pupate and emerged as *C. vicina*, *M. domestica*, and 1 unidentified species in the Calliphoridae family (Table). One *L. sericata* (Figure 1) and one *M. domestica* were caught alive in the house and kept in separate sterile containers. Both died during transport, but the *L. sericata* specimen laid eggs inside the container before dying.

Diagnostic Identification

The patient isolate grew on blood agar, yielding colonies with a smooth center and rough edges and displaying α hemolysis. With time, the colonies spread and exhibited a distinct brown pigment underneath. A brown pigment was also exhibited on heart infusion tyrosine agar. Moderate growth was recovered on MacConkey and phenylethyl alcohol agar. Optimal growth temperatures were 25°C and 35°C in aerobic conditions. No growth was recovered anaerobically or at 42°C. Colonies were strong oxidase positive, catalase positive. Acid was not produced from glucose, D-xylose, mannitol, lactose, sucrose, or maltose. Cells were nonmotile. Gram staining revealed large, pleomorphic, gram-negative rods, including curved and eye-shaped rods, some of which displayed vacuolation.

MALDI-TOF MS recorded a value of 2.40 for *W. chitiniclastica*, but the identification was presumptive because the MALDI-TOF MS database includes only *W. chitiniclastica* and the uniqueness of the isolate required confirmatory 16S rRNA sequencing.

Entomological Bacteriology

Six insect samples were inoculated on agar plates (Table, samples 2–7). *Wohlfahrtiimonas* sp. was isolated from 2 insect samples on blood agar plates incubated at 25°C. The first isolate was obtained from the fifth wash and diluted homogenate of the larva that hatched from the eggs laid by the green bottle fly. MALDI-TOF MS recorded a value of 2.41 and 2.43, respectively, using RUO library (unvalidated result). The second isolate was obtained from diluted homogenate of the pooled larvae collected from the carpet with a MALDI-TOF MS score of 2.29 (unvalidated result). These larvae were not taxonomically identified. The negative washes from these same samples demonstrated considerable bacterial overgrowth from which *Wohlfahrtiimonas* sp. could not be identified. *Wohlfahrtiimonas* sp. was not isolated from any other samples, including adult flies that emerged from the positive batch of larvae and from the fruit and meat fed to larvae and adult flies.

Isolate Relatedness

All isolates were successfully cut with *Not*I restriction enzyme. The PFGE pattern indicated that all isolates from flies and fly larvae were indistinguishable and were 74% similar to that of the patient isolate (Technical Appendix Figure 1).

References

1. Weyant RS, Moss CW, Weaver RE, Hollis DG, Jordan JJ, Cook EC, et al. Identification of unusual pathogenic gram-negative aerobic and facultatively anaerobic bacteria. 2nd ed. Baltimore, MD: Williams & Wilkins; 1996.

2. Bruker Daltonics Inc. MALDI Biotyper CA System user manual. Revision G. Document part number #603291. Billerica, MA: The Company; 2015.

3. Morey RE, Galloway RL, Bragg SL, Steigerwalt AG, Mayer LW, Levett PN. Species-specific identification of Leptospiraceae by 16S rRNA gene sequencing. J Clin Microbiol. 2006;44:3510–6. http://dx.doi.org/10.1128/JCM.00670-06

4. Saitou N, Nei M. The neighbor-joining method: a new method for reconstructing phylogenetic trees. Mol Biol Evol. 1987;4:406–25.

5. Felsenstein J. Confidence limits on phylogenies: an approach using the bootstrap. Evolution. 1985;39:783–91. http://dx.doi.org/10.1111/j.1558-5646.1985.tb00420.x

6. Tamura K, Nei M. Estimation of the number of nucleotide substitutions in the control region of mitochondrial DNA in humans and chimpanzees. Mol Biol Evol. 1993;10:512–26.

7. Tamura K, Stecher G, Peterson D, Filipski A, Kumar S. MEGA6: molecular evolutionary genetics analysis version 6.0. Mol Biol Evol. 2013;30:2725–9. http://dx.doi.org/10.1093/molbev/mst197

8. Borror DJ, Triplehorn CA, Johnson NF. Order Diptera. An introduction to the study of insects. Sixth ed. Chicago: Saunders College Publishing; 1989. p. 499–575.

9. CDC. Pictorial keys to arthropods, reptiles, birds, and mammals of public health significance. 2017 [cited 2017 Jan 19]; https://www.cdc.gov/nceh/ehs/publications/pictorial_keys.htm.

10. Whitworth T. Keys to the genera and species of blow flies (Diptera: Calliphoridae) of America north of Mexico. Proc Entomol Soc Wash. 2006;108:689–725.

11. Davies MP. Isolation and characterisation of bacteria associated with flying insects in hospitals, with particular emphasis on *Clostridium difficile*. PhD thesis, Aston University, 2014 [cited 2017 April 1]. http://eprints.aston.ac.uk/25334/

12. CDC. Standard operating procedure for PulseNet PFGE of *Escherichia coli* O157:H7, *Escherichia coli* non-O157 (STEC), *Salmonella* serotypes, *Shigella sonnei* and *Shigella flexneri*. 2013 [cited 2017 Apr 1]. https://www.cdc.gov/pulsenet/pdf/ecoli-shigella-salmonella-pfge-protocol-508c.pdf.

13. Tóth EM, Schumann P, Borsodi AK, Kéki Z, Kovács AL, Márialigeti K. *Wohlfahrtiimonas chitiniclastica* gen. nov., sp. nov., a new gammaproteobacterium isolated from *Wohlfahrtia magnifica* (Diptera: Sarcophagidae). Int J Syst Evol Microbiol. 2008;58:976–81. http://dx.doi.org/10.1099/ijs.0.65324-0

14. Barker HS, Snyder JW, Hicks AB, Yanoviak SP, Southern P, Dhakal BK, et al. First case reports of *Ignatzschineria (Schineria) indica* associated with myiasis. J Clin Microbiol. 2014;52:4432–4. http://dx.doi.org/10.1128/JCM.02183-14

15. Le Brun C, Gombert M, Robert S, Mercier E, Lanotte P. Association of Necrotizing Wounds Colonized by Maggots with Ignatzschineria-Associated Septicemia. Emerg Infect Dis. 2015;21:1881–3. http://dx.doi.org/10.3201/eid2110.150748

16. Roudiere L, Jean-Pierre H, Comte C, Zorgniotti I, Marchandin H, Jumas-Bilak E. Isolation of *Schineria* sp. from a man. Emerg Infect Dis. 2007;13:659–61. http://dx.doi.org/10.3201/eid1304.061255

17. Maurin M, Delbano JN, Mackaya L, Colomb H, Guier C, Mandjee A, et al. Human infection with *Schineria iarvae.* Emerg Infect Dis. 2007;13:657–9. http://dx.doi.org/10.3201/eid1304.061151

18. Zhou W, Li M, Zhu L, Hua F, Ji X, Sun Y, et al. Complete Genome Sequence of Wohlfahrtiimonas chitiniclastica Strain BM-Y, Isolated from the Pancreas of a Zebra in China. Genome Announc. 2016;4:e00643-16. http://dx.doi.org/10.1128/genomeA.00643-16

19. Diaz-Delgado J, Eva S, Isabel VA, Lucas D, Marisa A, Manuel A, et al. Endocarditis associated with *Wohlfahrtiimonas chitiniclastica* in a short-beaked common dolphin (*Delphinus delphis*). J Wildl Dis. 2015;51:283–6. http://dx.doi.org/10.7589/2014-03-072

20. Thaiwong T, Kettler NM, Lim A, Dirkse H, Kiupel M. First report of emerging zoonotic pathogen *Wohlfahrtiimonas chitiniclastica* in the United States. J Clin Microbiol. 2014;52:2245–7. http://dx.doi.org/10.1128/JCM.00382-14

21. Qi J, Gao Y, Wang GS, Li LB, Li LL, Zhao XM, et al. Identification of *Wohlfahrtiimonas chitiniclastica* isolated from an infected cow with hoof fetlow, China. Infect Genet Evol. 2016;41:174–6. http://dx.doi.org/10.1016/j.meegid.2016.04.008

22. Suryalatha K, John J, Thomas S. *Wohlfahrtiimonas chitiniclastica*-associated osteomyelitis: a rare case report. Future Microbiol. 2015;10:1107–9. http://dx.doi.org/10.2217/fmb.15.44

23. Rebaudet S, Genot S, Renvoise A, Fournier P-E, Stein A. *Wohlfahrtiimonas chitiniclastica* bacteremia in homeless woman. Emerg Infect Dis. 2009;15:985–7. http://dx.doi.org/10.3201/eid1506.080232

24. Nogi M, Bankowski MJ, Pien FD. *Wohlfahrtiimonas chitiniclastica* infections in 2 elderly patients, Hawaii, USA. Emerg Infect Dis. 2016;22:567–8. http://dx.doi.org/10.3201/eid2203.151701

25. Kõljalg S, Telling K, Huik K, Murruste M, Saarevet V, Pauskar M, et al. First report of *Wohlfahrtiimonas chitiniclastica* from soft tissue and bone infection at an unusually high northern latitude. Folia Microbiol (Praha). 2015;60:155–8. http://dx.doi.org/10.1007/s12223-014-0355-x

26. de Dios A, Jacob S, Tayal A, Fisher MA, Dingle TC, Hamula CL. First report of *Wohlfahrtiimonas chitiniclastica* isolation from a patient with cellulitis in the United States. J Clin Microbiol. 2015;53:3942–4. http://dx.doi.org/10.1128/JCM.01534-15

27. Campisi L, Mahobia N, Clayton JJ. *Wohlfahrtiimonas chitiniclastica* bacteremia associated with myiasis, United Kingdom. Emerg Infect Dis. 2015;21:1068–9. http://dx.doi.org/10.3201/eid2106.140007

28. Almuzara MN, Palombarani S, Tuduri A, Figueroa S, Gianecini A, Sabater L, et al. First case of fulminant sepsis due to *Wohlfahrtiimonas chitiniclastica.* J Clin Microbiol. 2011;49:2333–5. http://dx.doi.org/10.1128/JCM.00001-11

29. Schröttner P, Rudolph WW, Damme U, Lotz C, Jacobs E, Gunzer F. *Wohlfahrtiimonas chitiniclastica*: current insights into an emerging human pathogen. Epidemiol Infect. 2017;145:1292–303. http://dx.doi.org/10.1017/S0950268816003411

Technical Appendix Table. Summary of reported cases of *Wohlfahrtiimonas chitiniclastica* and the closely related *Ignatzschineria* species (syn. *Schineria* species) isolated among mammals worldwide

Species	Notable clinical presentation or postmortem findings	Organism isolated	Location sampled	Larvae present	Country	Reference
Human	Nonhealing, necrotic ulcers	*I. indica*	Blood	Yes, species unspecified	United States	(*14*)
Human	Nonhealing ulcers, urinary tract infection	*I. indica*	Urine	None	United States	(*14*)
Human	Severe lacerations	*I. indica**	Blood	Third instar larvae of *Lucila* (*Phaenicia) sericata*	United States	(*14*)
Human	Shoulder wound, hypotension with auricular fibrillation, cardiorespiratory arrest	*I. ureiclastica*	Blood	Yes, species unspecified	France	(*15*)
Human	Maceration of feet	*Schineria larvae* (syn. *I. larvae*)	Blood (only bacteria isolated)	Yes, species unspecified	France	(*16*)
Human	Maceration of feet (sale case as above, reinfection)	*S. larvae** (syn. *I. larvae*)	Blood	Yes, species unspecified	France	(*16*)
Human	Cutaneous ulcers, fever	*S. larvae* (syn. *I. larvae*)	Blood	Yes, species unspecified	France	(*17*)
Zebra	Unspecified	*W. chitiniclastica*	Pancreas	Unspecified	China	(*18*)
Dolphin	Found dead, endocarditis, septicemia	*W. chitiniclastica*	Blood, cardiac lesions	Unknown	Canary Islands	(*19*)
White-tailed deer	Found dead, tongue necrosis, septicemia	*W. chitiniclastica*	Tongue, liver	Unknown	United States (Michigan)	(*20*)
Cow	Cellulitis, purulence	*W. chitiniclastica*	Hoof crown	Unknown	China	(*21*)
Human	Deep ulcer, cellulitis, osteomyelitis	*W. chitiniclastica*	Ulcer	Unknown	India (Kerala)	(*22*)
Human	Superficial ulcers, neutropenia, febrile	*W. chitiniclastica**	Blood	Yes, not identified	France	(*23*)
Human	Umbilical and foot wounds, septic shock	*W. chitiniclastica*	Blood	Yes, not identified	United States (Hawaii)	(*24*)
Human	Decubitus ulcers	*W. chitiniclastica*	Deep wound	None	United States (Hawaii)	(*24*)
Human	Gangrene of distal leg	*W. chitiniclastica*	Surgically resected bone	None	Estonia	(*25*)
Human	Superficial ulcers	*W. chitiniclastica*	Wound swab	None	United States	(*26*)
Human	Skin excoriation	*W. chitiniclastica*	Blood	Third instar larvae of *Lucila* (*Phaenicia) sericata*	United Kingdom	(*27*)
Human	Occlusive peripheral arteriopathy of lower limbs, probable septic shock	*W. chitiniclastica**	Blood	None	Argentina	(*28*)
Human	Ulcers	*W. chitiniclastica*	Ulcer	None	Germany	(*29*)
Human	Ulcers	*W. chitiniclastica*	Ulcer	None	Germany	(*29*)
Human	Ulcers	*W. chitiniclastica*	Ulcer	None	Germany	(*29*)
Human	Leg ulcer	*W. chitiniclastica*	Ulcer	None	Germany	(*29*)

*Blood samples for which *W. chitiniclastica* or *Ignatzschineria* spp. were the only organisms isolated.

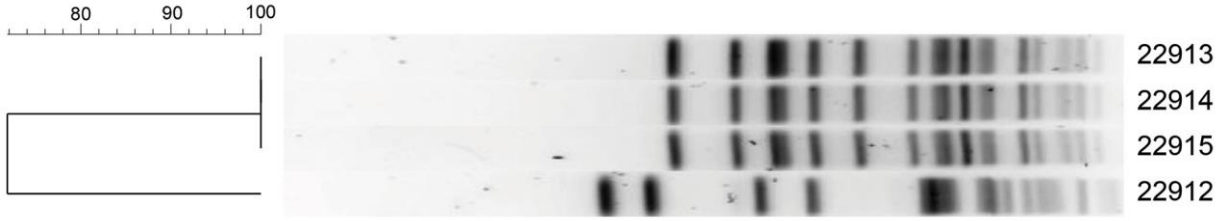

Technical Appendix Figure. Dendrogram of pulsed-field gel electrophoresis patterns of isolates from flies and fly larvae, Washington, USA. From top to bottom: batch of larvae (diluted homogenate); *Lucila sericata* larvae (fifth wash); *L. sericata* larvae (diluted homogenate); and patient isolate.
